# Sugar-Responsive Pseudopolyrotaxane Composed of Phenylboronic Acid-Modified Polyethylene Glycol and γ-Cyclodextrin

**DOI:** 10.3390/ma8031341

**Published:** 2015-03-20

**Authors:** Tomohiro Seki, Misato Namiki, Yuya Egawa, Ryotaro Miki, Kazuhiko Juni, Toshinobu Seki

**Affiliations:** Faculty of Pharmaceutical Sciences, Josai University, Keyakidai, Sakado, Saitama 350-0295, Japan; E-Mails: gyd1203@josai.ac.jp (T.S.); yy09237@josai.ac.jp (M.N.); rmiki@josai.ac.jp (R.M.); kjuni@josai.ac.jp (K.J.); sekt1042@josai.ac.jp (T.S.)

**Keywords:** pseudopolyrotaxane, cyclodextrin, boronic acid, drug delivery, stimuli-responsive material

## Abstract

We have designed a sugar-responsive pseudopolyrotaxane (PPRX) by combining phenylboronic acid-modified polyethylene glycol (PBA–PEG) and γ-cyclodextrin. Phenylboronic acid (PBA) was used as a sugar-recognition motif in the PPRX because PBA reacts with a diol portion of the sugar molecule and forms a cyclic ester. When D-fructose or D-glucose was added to a suspension of PPRX, PPRX disintegrated, depending on the concentration of the sugars. Interestingly, catechol does not show a response although catechol has a high affinity for PBA. We analyzed the response mechanism of PPRX by considering equilibria.

## 1. Introduction

Cyclodextrins (CyDs) are enzymatically produced cyclic oligosaccharides composed of glucopyranoside units. CyDs composed of six, seven, or eight D-glucose (Glc) units are named as α-, β-, or γ-CyD, respectively, and are widely used [1]. The torus shape of CyDs enables the formation of inclusion complexes between CyDs and guest molecules via hydrophobic interactions. Guest molecules are not only limited in low molecular weight compounds but also include polymers such as polyethylene glycol (PEG) [2,3]. Harada *et al.* first developed a supramolecular structure composed of a PEG chain and many α-CyDs [2]. Such a structure is called a pseudopolyrotaxane (PPRX), known as a molecular necklace, and this unique structure has attracted much attention [4,5,6,7,8]. The combination of γ-CyD and PEG provides a PPRX in which two PEG chains penetrate many cavities of γ-CyD [3].

Formulators have attempted to apply PPRXs for drug delivery systems on the basis of disintegration of PPRX [6]. For example, Higashi *et al.* have reported using PPRXs for sustained release of PEGylated proteins [9,10]. Using PEGylated protein is a reasonable approach because PEGylation sometimes improves the efficacy of protein drugs [11,12]. PPRX composed of PEGylated insulin and CyD showed an extended hypoglycemic effect [9]. Ohya *et al.* have prepared a supramolecular structure composed of drug-modified CyDs, PEG, and bulky end-caps at the terminals of PEG with a hydrolyzable peptide linkage [13]. Hydrolysis of the end-capped linkages by a protease induced disintegration of PPRX, resulting in the release of drug-modified CyDs. This is a kind of stimulus-sensitive material for drug delivery systems.

**Figure 1:**
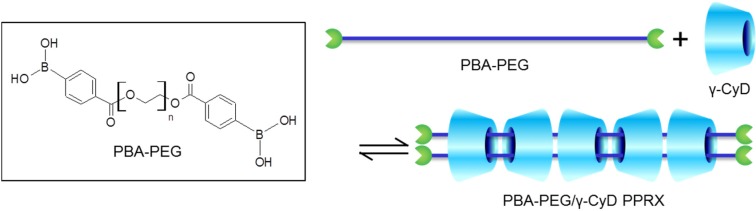
Chemical structure of phenylboronic acid-modified polyethylene glycol (PBA–PEG) and schematic illustration of PEG-PBA/γ-cyclodextrin pseudopolyrotaxane (γ-CyD PPRX).

In this study, we fabricated a sugar-responsive PPRX using phenylboronic acid-modified PEG (PBA–PEG in Figure 1). PBA reversibly reacts with a diol functional group of sugars to form a five- or six-membered ring via ester bonds [14]. Consequently, PBA derivatives have been widely studied as sugar-recognition motifs in chemical probes for sugar [15,16,17,18]. Furthermore, researchers are quite interested in developing a sugar-responsive insulin-release system on the basis of PBA properties [19,20,21]. Insulin self-injection treatments involve difficulties of controlling the blood sugar level and risk of hypoglycemia [22]. Sugar-responsive insulin-release systems will contribute to overcome the difficulties of insulin treatments. Developing the sugar-responsive PPRX-containing PEG will facilitate the fabrication of new sugar-induced PEGylated insulin-release systems.

Although CyDs are oligosaccharides, PBA does not bind CyDs because CyDs do not have cis-diols. Some reports showed intermolecular interaction between PBA derivatives and cavities of CyDs, but did not show the formation of covalent cyclic ester bonds between PBA and CyDs [23,24,25,26]. Therefore, we expected the formation of PBA–PEG/γ-CyD PPRX through intermolecular interactions.

## 2. Results and Discussion

### 2.1. Preparation of PBA–PEG/γ-CyD PPRX

We modified PBA in two terminals of PEG (molecular weight (MW) 2000), and PBA–PEG was applied to a preparation of PPRX. In order to confirm the existence of an intermolecular interaction between PBA–PEG and γ-CyD, we monitored ^1^H NMR of D_2_O solution containing PBA–PEG (0.44 mM) and γ-CyD (0–20 mM). The chemical shift of PEG chain was observed at 3.52 ppm, and it moved to lower field depending on the concentration of γ-CyD, demonstrating the complexation between PEG and γ-CyD (Figure S1).

From an aqueous solution of PBA–PEG and γ-CyD, white precipitates were obtained as PBA–PEG/γ-CyD PPRX. In a similar manner, we tried to obtain PBA–PEG/α-CyD PPRX; however, the resulting precipitate did not contain the PBA moiety, which implies that α-CyD accelerates the hydrolysis of the carboxyester between PBA and PEG [27]. For that reason, α-CyD was not used in the following experiments.

**Figure 2:**
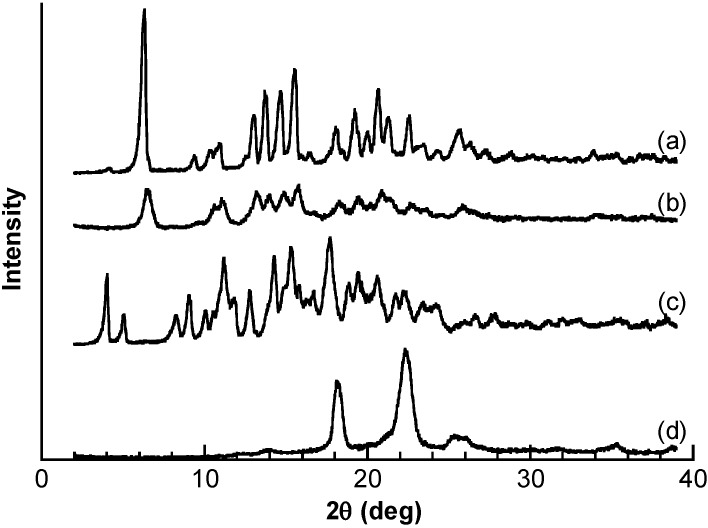
XRD patterns: (**a**) PEG/γ-CyD PPRX; (**b**) PBA–PEG/γ-CyD PPRX; (**c**) γ-CyD; (**d**) PBA–PEG.

Both differential scanning calorimetry (DSC) and powder X-ray diffraction (XRD) were used to investigate the solid state of the resulting PBA–PEG/γ-CyD PPRX. In the DSC thermographs of PBA–PEG, an endothermic peak was observed at 53 °C, corresponding to the melting point of PBA–PEG (Figure S2). In the case of PBA–PEG/γ-CD PPRX, the endothermic peak completely disappeared, which demonstrated that PBA–PEG is fully included in the cavities of γ-CyD [28]. XRD pattern of PBA–PEG/γ-CyD PPRX was consistent with that of PEG/γ-CyD (Figure 2), suggesting that PBA–PEG/γ-CyD PPRX complex contains a double-stranded PEG [10].

To confirm the stoichiometry of the components of PPRX, we dissolved PBA–PEG/γ-CyD PPRX into dimethyl sulfoxide (DMSO-*d*_6_) and measured the solution with the single-proton nuclear magnetic resonance (^1^H NMR). The spectrum showed that the stoichiometry between the ethylene glycol unit and γ-CyD was 4:1, which was the same as PEG/γ-CyD [3]. These results of DSC, XRD, and ^1^H NMR analyses showed that PBA–PEG/γ-CyD was successfully obtained.

### 2.2. Sugar Response of PBA–PEG/γ-CyD PPRX

**Figure 3:**
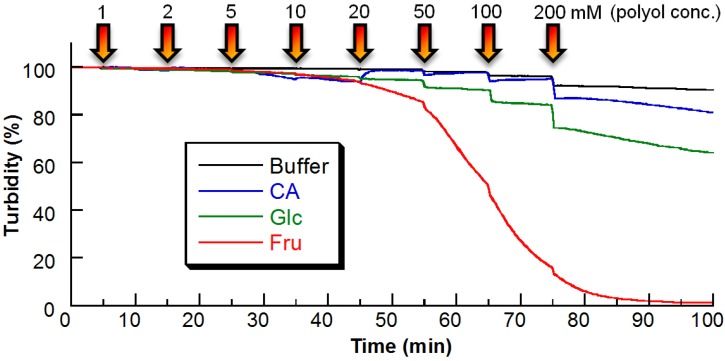
The turbidity change of the PBA–PEG/γ-CyD PPRX depending on the sugar concentration (pH 7.4, 37 °C).

Sugar responses of PBA–PEG/γ-CyD PPRX were studied by means of turbidity measurements (Figure 3). PBA–PEG/γ-CyD (23 mg) was suspended in a buffer solution (2.0 mL, 20 mM 2-[4-(2-hydroxyethyl)piperazin-1-yl]ethanesulfonic acid (HEPES) buffer, pH 7.4, 37 °C). After the turbidity became constant, a stock sugar solution was added to the suspended solution. The turbidity decreased as the sugar concentration increased, indicating that the solid of PPRX disintegrated and was dissolved by the effect of sugar. The effect of D-fructose (Fru) was larger than that of Glc. Figure 3 shows that the release rate was accelerated by the sugar addition, and the release speed depended on the kind and concentration of the sugars. As a control experiment, we confirmed that PEG/γ-CyD PPRX showed no response for Fru and Glc.

Furthermore, we recorded a response for catechol (CA), which has a higher affinity for PBA. According to published data, the binding constants of PBA at pH 7.4 to CA, Fru, and Glc are 830, 160, and 4.6 M^−1^, respectively [29]. Surprisingly, CA showed a negligible response despite its high affinity for PBA, indicating that the response of PBA–PEG/γ-CyD necklace is controlled not only by the affinity for PBA.

### 2.3. Response Mechanism of PBA–PEG/γ-CyD PPRX

**Figure 4:**
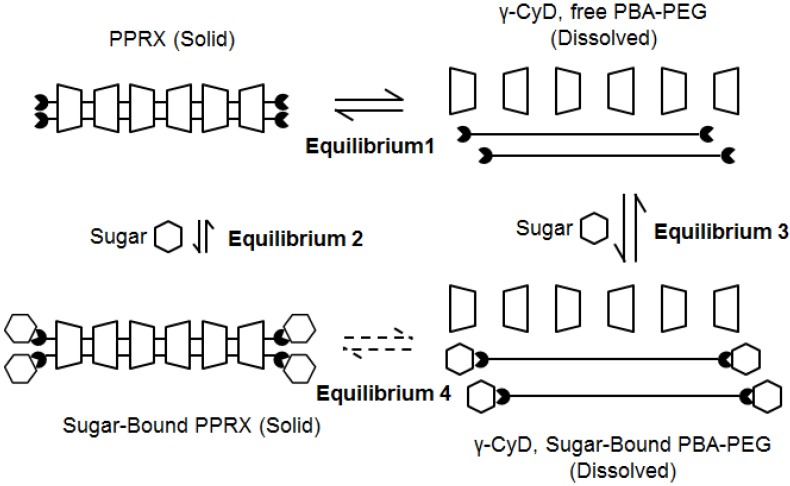
Proposed mechanism of sugar-induced disintegration of PPRX.

To explain the response mechanism of PPRX to polyols, we proposed the following scheme of equilibria (Figure 4): When PPRX was suspended in an aqueous solution, some amount of PBA and PEG were dissolved in the solution. In other words, there was solubility equilibrium (Equilibrium 1 in Figure 4). When the sugar (Glc or Fru) was added, the solid-state PPRX may directly bind the sugar; however, the binding occurs only on the solid surface. Thus, the reversible reaction between the solid-state PPRX and the sugar is slow and its contribution to the sugar response of PPRX is quite small (Equilibrium 2). The dissolved PBA–PEG binds the sugar and forms a cyclic ester (Equilibrium 3). Because the sugar is relatively bulky and hydrophilic, the sugar-PBA ester hardly penetrates the cavities of γ-CyD, which means the sugar-bound PPRX is not obtained from the sugar bound PBA–PEG. Even though the sugar-bound PPRX was formed, the end-capping effect of Glc interferes with disintegration. Accordingly, Equilibrium 4 does not exist.

On the basis of these proposed equilibria, the effect of sugar can be explained as follows: The sugar addition decreases free PBA–PEG, which induces the further disintegration of PPRX to compensate for the lack of free PBA–PEG. The sugar-bound PBA–PEG does not contribute to the sugar-bound PPRX because the bulky sugar does not pass through γ-CyD cavities. In total, the solid-state PPRX decreases following the sugar addition. Fru induced the remarkable decrease in the turbidity experiment compared to Glc because Fru has a higher affinity for PBA–PEG and efficiently reduces free PBA–PEG, which causes the faster disintegration of PPRX.

The limited response of CA to PPRX disintegration is described as follows. In a similar manner, the free PBA–PEG binds CA. Because the size of CA is similar to that of PBA, CA–PBA ester can pass thorough γ-CyD; *i.e.*, Equilibrium 4 exists in the case of CA. Even though PPRX disintegrates, CA-bound PPRX regenerates from CA-bound PBA–PEG. Consequently, CA does not affect the amount of the solid.

We experimentally confirmed that CA is bound to PBA terminals. PPRX (70 mg) was suspended in an aqueous CA solution (23 mM, 0.35 mL). After 21 h, the solid was filtered and dried (53 mg), and then the recovered PPRX was dissolved in DMSO-*d*_6_ to obtain its ^1^H NMR spectrum. In the spectrum, peaks derived from CA were observed, and their integrated values indicated that over twenty percent of PBA bound CA in the recovered PPRX. In case of a Fru solution instead of the CA solution, peaks derived Fru was not observed in the ^1^H NMR spectrum of recovered PPRX, showing that the direct binding between the solid state PPRX and polyols hardly occurs. To explain the result in the case of CA, we suggest that PBA–PEG dissolves once in water and binds CA. Finally, CA-bound PBA–PEG and γ-CyD form CA-bound PPRX. These experimental results prove the validity of the proposed equilibria.

In order to gain further insight about the proposed equilibria, we prepared PPRX in the presence of Fru or CA, and investigated the components of PPRX with ^1^H NMR. The obtained PPRX in the presence of Fru did not contain Fru. This result shows that Equilibrium 2 is not important. Furthermore, Equilibrium 4 does not exist in the case of Fru. In contrast, the PPRX obtained in the presence of 200 mM CA involved CA, proving the presence of Equilibrium 4 in the case of CA. These results also support our proposed equilibria in Figure 3. In the case of Fru or Glc, there is not Equilibrium 4, which plays a key role in the sugar responsiveness of PBA–PEG/γ-CyD PPRX.

## 3. Experimental Section

### 3.1. Materials

α-CyD and γ-CyD were purchased from Junsei Chemical Co., Ltd. (Tokyo, Japan). Polyethylene glycol (MW 2000), *p*-carboxyphenylboronic acid, thionyl chloride, and 2,2-dimethyl-1,3-propanediol were obtained from Wako Pure Chemical Industries (Osaka, Japan). HEPES was purchased from Dojindo Laboratories (Kumamoto, Japan). All other chemicals were of reagent grade and were used as received.

### 3.2. Apparatus

^1^H NMR spectra were recorded with a Varian 400-MR (Agilent Technologies, Santa Clara, CA, USA). Turbidity was monitored with a V-530 UV–vis spectrometer (JASCO Corporation, Tokyo, Japan) with absorbance at 700 nm. DSC was conducted with a Thermo Plus2 series (Rigaku Corporation, Tokyo, Japan). The sample was heated in an aluminum pan under a nitrogen atmosphere at a heating rate of 5 K/min. Powder XRD patterns were measured by a Mini FlexII (Rigaku Corporation) under these conditions: CuKα radiation and diffraction were done at 30 kV, 15 mA with a scanning speed of 4°/min, and a measurement range of 2θ = 2°–39°. Matrix-assisted laser desorption/ionization time-of-flight mass spectrometry (MALDI-TOF MS) spectra were recorded with AXIMA-CFR plus (Shimadzu, Kyoto, Japan).

### 3.3. Preparation

#### 3.3.1. Synthesis of PBA–PEG

*p*-Carboxyphenylboronic acid (1.60 g, 9.64 mmol) and 2,2-dimethyl-1,3-propanediol (1.19 g, 11.6 mmol) were dissolved into tetrahydrofuran and refluxed for one day to protect the boronic acid moiety. The solvent was evaporated and the residue was dissolved in thionyl chloride (20.0 mL, 275 mmol), and three drops of dimethylformamide (DMF) was added to the solution and stirred for 6 h at 74 °C. DMF and thionyl chloride were evaporated, and the residue contained *p*-chlorocarbonylboronic acid and 2,2-dimethyl propanediol-1,3 cyclic ester. The residue and PEG (MW 2000, 1.96 g) were dissolved in a mixed solution of anhydrous dichloromethane (20.0 mL) and anhydrous pyridine (1.0 mL). It was stirred at 0 °C under a nitrogen atmosphere for one day. The solvent was evaporated, and water (100 mL) was added to the residue. The solution was filtered with a glass filter to remove the insoluble matter, and the filtrate was dialyzed against water using a dialysis tube (MWCO 1000). The resulting solution was lyophilized, and PBA–PEG was obtained (843 mg, 43.7%). Analytical data were as follows:

^1^H NMR (400 MHz, DMSO-*d*_6_) δ 8.29–8.28 (s, 4H, BOH), 7.91–7.89 (s, 8H, phenyl-H), 3.51–3.48 (m, 180H, PEG).

MALDI-TOF MS *m/z*: 2254.81 [M + H]^+^.

#### 3.3.2. Preparation of PBA–PEG/γ-CyD PPRX

PBA–PEG (31.4 mg, 13.9 μmol) and γ-CyD (200 mg, 154 μmol) were dissolved in water (1.00 mL), and the solution was kept at room temperature. After three days, the resulting precipitate of the molecular necklace was filtered and dried under reduced pressure (214 mg).

PBA–PEG/γ-CyD molecular necklace was dissolved in DMSO-*d*_6_ and measured with ^1^H NMR. When the integration of H-1 of CyD was set to 8.00, the integration of PEG part was 16.0. From this result, we calculated the stoichiometry between the ethylene glycol monomer unit and γ-CyD to be 4.0:1.0.

### 3.4. Turbidity Measurements

A buffer solution (20 mM HEPES, pH 7.4, 2.0 mL) in a cell for an absorption spectrometer was stirred at 37 °C. PBA–PEG/γ-CyD PPRX (23 mg) was added to and suspended in the stirred buffer solution. The turbidity was recorded with absorbance at 700 nm. After a while, the turbidity reached a constant, and a small amount of stock sugar solution (1.00 M) was added at 10-min intervals to the suspended solution to increase the sugar concentration.

## 4. Conclusions

We have successfully designed a novel stimuli-responsive PPRX. The modified PBA at the terminal of PEG acts as a sugar-recognition motif. The binding between PBA moiety and the sugar induced a disintegration of PPRX. We explained the sugar response by using equilibrium movements. To apply this PPRX for a PEGylated insulin delivery system, we should improve the affinity of PBA moiety for Glc. Current guidelines recommend a post-meal Glc level of <10 mM and a fasting plasma Glc level of 3.9–7.2 mM [22]. At present, some reports suggest that certain bis-boronic acid derivatives show a high affinity for Glc [30,31,32,33]. The introduction of a well-designed bis-boronic acid derivative to the terminal PEG perhaps will be promising.

## Supplementary Materials

Supplementary materials can be accessed at: http://www.mdpi.com/1996-1944/8/3/1341/s1.
