# Supplementary material for: Sugar-Responsive Pseudopolyrotaxane Composed of Phenylboronic Acid-Modified Polyethylene Glycol and γ-Cyclodextrin
Source: Materials (Basel). 2015 Mar 20;8(3):1341–9. doi: 10.3390/ma8031341 (PMC5455434; doi:10.3390/ma8031341)
Supplement: Supplementary file 1 [file materials-08-01341-s001.pdf]

Supplementary Materials

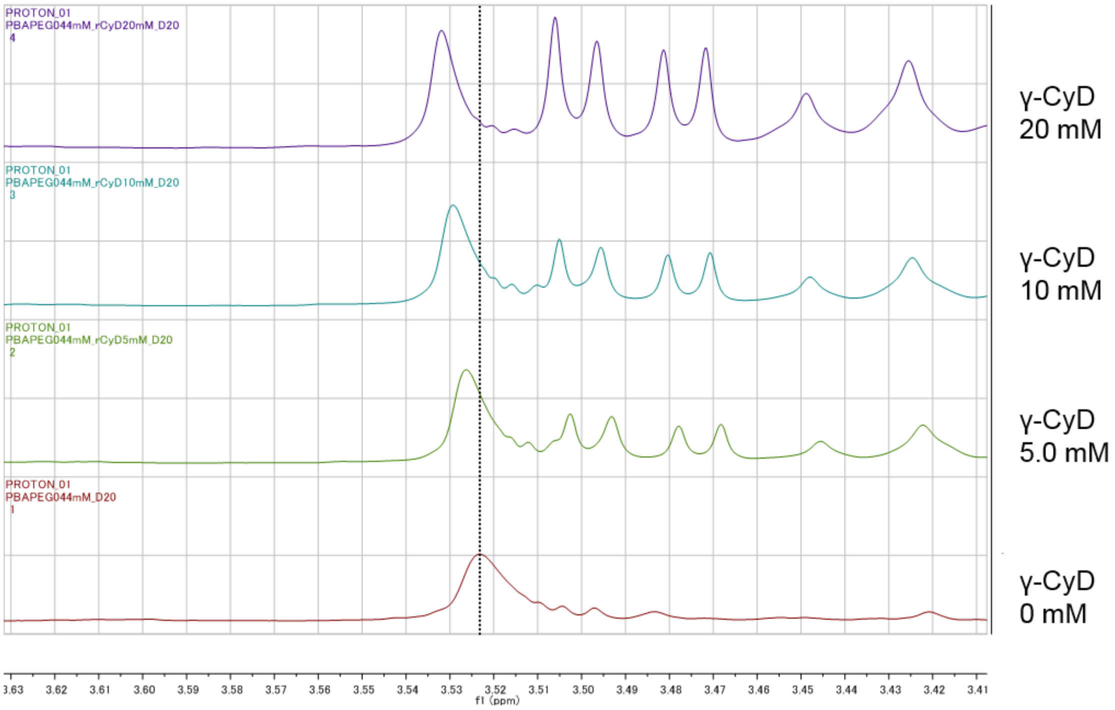

Figure S1. The ^{1}H NMR spectra of PBA–PEG (0.44 mM) with various concentrations of γ-CyD (0, 5.0, 10, 20 mM) in D_2O.

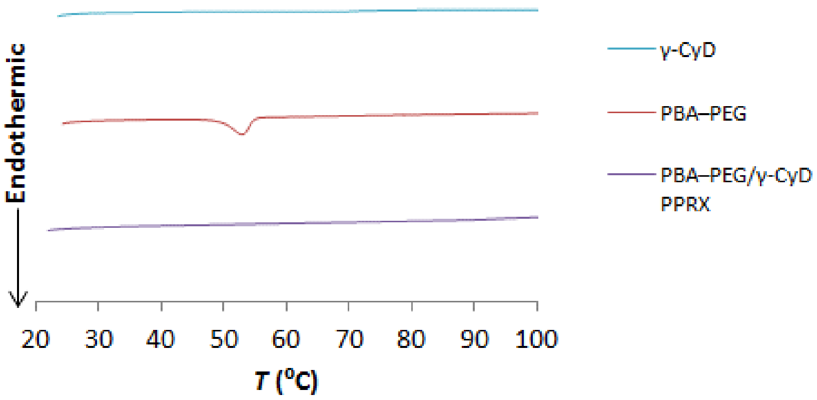

Figure S2. DSC thermograms of γ-CyD, PBA–PEG, and PBA–PEG/γ-CyD PPRX.
